# Histamine Intolerance: Symptoms, Diagnosis, and Beyond

**DOI:** 10.3390/nu16081219

**Published:** 2024-04-19

**Authors:** Christoph Jochum

**Affiliations:** Charité-Universitätsmedizin Berlin, Corporate Member of Freie Universität Berlin and Humboldt Universität zu Berlin, Department of Hepatology and Gastroenterology, Campus Charité Mitte and Campus Virchow Klinikum, 10117 Berlin, Germany; christoph.jochum@charite.de

**Keywords:** histamine intolerance, food intolerance, histamine, diamine oxidase (DAO)

## Abstract

Histamine intolerance is a condition characterized by the accumulation of histamine to a point that exceeds the body’s capacity to eliminate it. Researchers have attributed several reasons to this condition, such as genetic factors, alcohol, and dietary deficiencies, among other elements. Symptoms of histamine intolerance have been found to extend beyond the gastrointestinal tract and to the whole body, with these symptoms being sporadic and non-specific. This review will explore various aspects related to histamine intolerance, such as its causes, symptoms, diagnosis, and information related to management.

## 1. Introduction

Food intolerance refers to a negative reaction to a food or one of its components, occurring at a level that would normally be harmless, and not involving an immune system response [1]. In the United States, the results of a recent survey that included 2133 participants indicated that the prevalence of food intolerance was approximately 25% among survey respondents [2]. Similarly, research suggests that food intolerance affects about one in five individuals worldwide, highlighting the significance of the condition [1,3]. It is important not to confuse food intolerance with food allergy, which is a less prevalent (compared to food intolerance) immune-mediated response to the protein component of food [1]. Several contributing factors lead to food intolerance, including histamine accumulation.

Histamine is a biogenic amine with several biological effects across different types of cells mediated through the activation of histamine receptors [4]. Histamine receptors are categorized into four classes: H1 up to H4. Each type of these receptors has a different signaling pathway that triggers a different reaction. In the gastrointestinal tract, histamine concentrations are relatively high, with H1 and H2 receptor subtypes being the main classes present in that digestive pathway [4]. Their effect is more evident during inflammatory responses and similar reactions. Histamine exerts its effects by acting on cells, neurons, smooth muscle, and others. Histamine is also present in various types of food, such as cheese, wine, fermented foods, spinach, some fish types, meat, and others [5].

Histamine intolerance occurs when the body’s capacity to eliminate histamine is exceeded by the rate of histamine accumulation. In healthy individuals, intestinal diamine oxidase (DAO), a class of enzymes, helps eliminate histamine taken from food [4]. When the activity of DAO is inhibited by factors such as medications, or its expression is reduced due to internal factors such as genetic mutations, the body’s ability to manage histamine is significantly affected [4]. This leads to the accumulation of histamine, causing symptoms that, among others, mimic allergic reactions. For example, about 20% of the European population consumes medications that could decrease DAO activity, increasing the risk of histamine intolerance [4,6]. Examples of drugs that have been found to affect DAO’s activity include verapamil, clavulanic acid, and isoniazid, among others.

In addition to the above, researchers found that several other factors could contribute to histamine intolerance. In this context, alcohol was found to increase the release of endogenous histamine, affecting the rate of its degradation due to the presence of excess biogenic amine [4]. Other elements that have been found to factor into the equation of histamine intolerance by decreasing DAO activity include mineral and vitamin deficiencies, such as vitamin C and copper. Moreover, the menstrual cycle has also been found to contribute to this phenomenon [4].

In this review, we will discuss food intolerance with more focus on histamine intolerance, providing an overview of the condition, causative factors, symptoms, diagnosis, and possible management paths.

## 2. Histamine Intolerance Etiology

**Figure 1:**
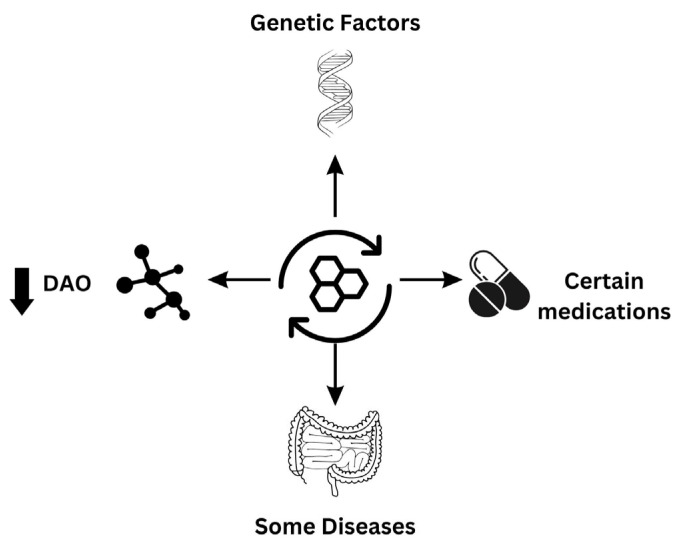
Major factors contributing to the etiology of histamine intolerance.

As discussed, the DAO enzyme helps control and protect against high levels of exogenous histamine ingested through food. Figure 1 summarizes the major factors that have been attributed to drive the condition. The available clinical literature indicates that individuals displaying histamine intolerance symptoms have lower levels of plasma DAO [6,7]. This highlights the importance of the DAO enzyme as a protective factor against histamine intolerance and demonstrates how a deficiency in this enzyme due to genetic, pathological, or pharmacological factors predisposes individuals to the condition [6,8].

Concerning the genetic component of histamine intolerance, researchers have analyzed the polymorphism in genes encoding enzymes related to histamine metabolism, like DAO and l-histidine decarboxylase, among others [6]. The researchers identified over 50 single-nucleotide polymorphisms in the gene encoding DAO, with some of these polymorphisms producing a protein product that has been shown to have an altered activity that promotes histamine intolerance symptoms [6,9,10,11,12]. These studies highlighted several genetic polymorphisms with the most significant effects on DAO enzyme activity. In addition, these studies mentioned that specific genetic polymorphisms play a more prominent role in certain ethnicities.

Just as researchers identified genetic polymorphisms that inhibit DAO activity, studies have shown several other genetic variations that enhance the enzyme’s activity [13]. Although the presented results are inconclusive, it is worth mentioning that the literature indicates that patients suffering from diseases affecting the gastrointestinal, cardiovascular, and nervous systems, among others, have been found to have mutations influencing DAO activity in their bodies [6,10]. However, whether the outcome is positive or negative requires further investigation.

In addition to the genetic component, DAO deficiency could result from medications or certain diseases. For example, patients suffering from inflammatory bowel disease have been found to have suppressed DAO activity in a way that corresponds to mucosal damage [14,15]. This indicates a possible value for a marker of gastrointestinal mucosal integrity. Furthermore, DAO deficiency has been observed in certain functional diseases related to the gastrointestinal tract, like carbohydrate malabsorption [6,16,17]. In this regard, Griauzdaite et al. [17] found that 9 out of 10 patients with nonceliac gluten sensitivity (NCGS) have reduced serum DAO levels, indicating a potential relationship between NCGS and histamine intolerance.

Other factors like alcohol and certain medications have been observed to cause a temporary and reversible DAO deficiency [18]. This results in excess histamine levels entering systemic circulation, causing symptoms similar to allergic reactions. Regarding medications, the blood-pressure-lowering medication verapamil has a potent inhibitory effect on DAO enzymatic activity [6,19].

## 3. Histamine Intolerance: Symptoms

**Figure 2:**
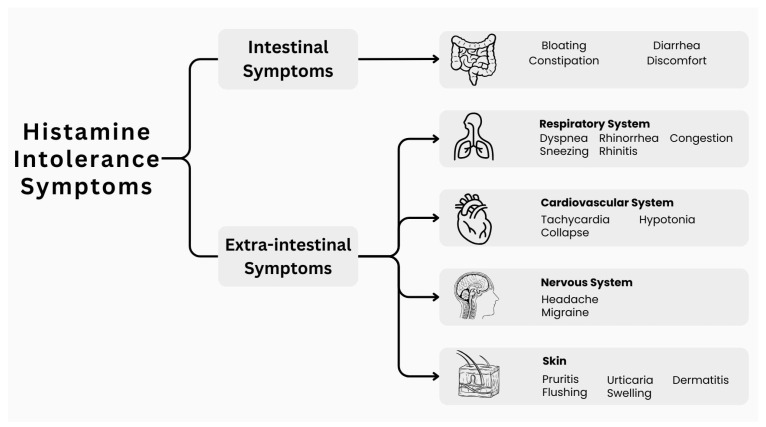
Symptoms associated with histamine intolerance.

The symptoms and manifestations of histamine intolerance are heterogenic and include intestinal and extraintestinal symptoms [19,20]. Figure 2 summarizes the diverse nature of histamine intolerance symptoms. These symptoms could be diverse and non-specific. This is because histamine intolerance is associated with excess histamine that enters the systemic circulation through the intestines, interacting with histamine receptors that are present in multiple locations across the body, which makes a typical clinical outline of symptoms challenging to achieve [21]. The presence of diverse, unexpected, and random symptoms, particularly after food consumption, suggests a strong possibility of histamine intolerance.

Histamine intolerance can cause a range of gastrointestinal symptoms, including bloating, abdominal discomfort, gas, diarrhea or constipation, and other related issues [6,19]. As mentioned earlier, there are other extraintestinal manifestations since histamine receptors are present across the body. For example, respiratory symptoms associated with histamine receptor intolerance include rhinorrhea, rhinitis, nasal congestion, dyspnea, and sneezing [19,21]. Histamine receptors are present in the skin; therefore, there will be skin manifestations, including pruritis, flushing, urticaria, dermatitis, and swelling. Other affected systems include the reproductive system (menstrual cramps), the cardiovascular system (tachycardia, hypotonia, and collapse), and the nervous system (headache and migraine) [6,19,21].

One important thing to note is that symptom manifestations in the latter three systems are less common [6,21]. In this context, a study by Schnedl et al. assessed the symptoms in a sample of 133 patients suffering from histamine intolerance [20]. The researchers found that the most frequent symptoms included gastrointestinal manifestations, with bloating being present in 92% of subjects. Other common symptoms included constipation, abdominal pain, diarrhea, and postprandial fullness, with proportions of 55%, 68%, 71%, and 73%, respectively [20]. When the subjects were asked to rate the severity on a scale from 1 to 5 (higher, more severe symptoms), they indicated bloating as the most severe symptom with a score of 4. Regarding skin manifestations, pruritis was the most common symptom and was reported by 48% of the subjects; similar results with respiratory system manifestations of rhinorrhea, nasal congestion, and sneezing were obtained [20]. Concerning the cardiovascular system, dizziness (66%), headache (65%), and palpitations (47%) were the most reported symptoms. Interestingly, a study by Wöhrl et al. on 10 healthy female subjects found that supplementing 75 mg of histamine, a dose present in typical meals, provoked symptoms that mimic histamine intolerance [22]. Regarding the latter study, the authors indicated that despite the volume of the dose being within a tolerable threshold, under normal settings, food would contain other ingredients, like water, glucose, and amino acids, which could affect the dose of available histamine. Nevertheless, the results of this study provide valuable insights.

Taken together, these findings demonstrate how diverse and complex are the clinical manifestations of histamine intolerance. In addition, they indicate that even normal doses given to healthy subjects could induce symptoms of histamine intolerance. However, further research is needed to confirm such results.

## 4. Diagnosis of Histamine Intolerance

Due to the diversity of the symptoms and their potential overlap with other diseases, in addition to the lack of consensus on a single diagnostic approach, there are no specific direct tests that can be used to determine histamine intolerance [19]. However, research indicates that since the condition is driven by factors that cause a decrease in the levels and/or activity of DAO enzymes, it could be a useful marker to detect the disease. Yet, its reference value to diagnose histamine intolerance has not been clearly defined [16,21]. Table 1 summarizes the diagnostic approaches utilized in histamine intolerance diagnosis.

In the case of serum DAO, enzyme-linked immunosorbent assay and other techniques could be used to quantify the amount of histamine degrading over a determined time threshold in a blood sample [21]. Although challenges related to DAO detection in the sample have led some researchers to question its validity, methods to determine its levels leveraging the said techniques have been developed, tested, and proved useful [23,24]. Among the disadvantages of serum DAO is its variability across different times of the day in the same person, as demonstrated by Pinzer et al. [25]. Therefore, it is recommended not to base the diagnosis on serum DAO solely but to use it with other diagnostic tests. Another approach that utilizes DAO is measuring its activity through a biopsy of the intestinal mucosa. However, further research is needed to confirm the robustness of this approach [6,21].

In addition to the above, the skin prick test has been suggested as an approach to determine histamine intolerance. In this test, the redness of the histamine control is resolved at times later than observed in healthy controls, indicating the body’s reduced capacity to eliminate histamine [21]. However, this test comes with limitations such as the inability of this test to discriminate between histamine intolerance and other allergic conditions [26]. In addition, intracutaneous histamine degradation might not accurately reflect its degradation rate in the intestines.

The histamine challenge test is another diagnostic approach that could be used to diagnose histamine intolerance. In addition to its diagnostic value, it has been determined that it can be used to identify individual tolerance limits [21]. In other words, it can be used to determine the dose that can trigger a response in susceptible individuals. Notably, despite the test’s value in determining the tolerance threshold, it is difficult to accurately quantify the amount of histamine present in food [18,27]. One of the drawbacks associated with this approach is the need for supervision by a specialist. In this test, subjects take 75 mg of histamine, which is considered harmless in healthy individuals. However, there have been reports of this dose producing side effects in healthy people [22].

Other methods to diagnose histamine intolerance have been suggested. However, due to applicability, their use is limited. For example, analyzing fecal histamine levels has been suggested as a diagnostic approach, but it is unreliable since intestinal microbiota represents a significant source of histamine [21].

Genetic evaluation is warranted in some instances since some individuals have a genetic tendency for histamine intolerance. Currently, researchers have identified several single-nucleotide polymorphisms that predispose individuals to histamine intolerance [10]. This test can be combined with other tests to confirm diagnosis in susceptible individuals.

Taken together, research shows that it is better to base the diagnosis on a combination of tests and medical history to avoid misdiagnosis due to the overlapping nature of the condition with several other diseases.

## 5. Management of Histamine Intolerance

There are multiple approaches to managing histamine intolerance, with a low-histamine diet being the gold standard [21]. The first approach is stopping medications that interact with DAO. Other techniques involve DAO supplementation to help degrade ingested histamine [6]. In more severe conditions, antihistamine medications may be dispensed, though their use is only recommended for a short period of time.

The low-dose histamine diet mainly revolves around avoiding foods that contain excessive amounts of histamine and biogenic amines. These foods include seafood, fermented soybean products, aged cheese, avocado, chocolate, nuts, milk, legumes, and fruits like bananas [19,21]. Examples of foods low in histamine (when used in normal quantities) include water, fresh juices, herbal teas, bread, rice, eggs, honey, and others. When using this approach, explaining to histamine-intolerant patients that these food groups could be reintroduced at later stages under certain quantities is important. In this context, research shows that patients who respond to this approach should continue it for a month until symptoms are no longer present [19]. Then, food is gradually reintroduced. When a low-dose histamine diet approach was used, gastrointestinal, cutaneous, and other manifestations were reported to improve, in addition to enhancing DAO levels when subjects were compliant with the diet [27,28,29].

Exogenous DAO supplementation is another proposed approach to control histamine intolerance. In this regard, several studies have shown positive results in terms of symptom improvement, antihistamine medication use, serum DAO increase, and other beneficial outcomes [30,31,32,33,34]. In their study, Komericki et al. evaluated the impact of oral DAO supplementation (provided as capsules containing 10,000 histamine degrading units, among other ingredients) on 39 patients who displayed histamine intolerance symptoms, like headache, skin, mucous membranes, and gastrointestinal symptoms, upon oral provocation test [30]. Their results revealed that when compared to the placebo, DAO supplementation resulted in a statistically significant improvement in histamine intolerance symptoms. The study by Schnedl et al., which lasted for 8 weeks, evaluated 28 patients suffering from histamine intolerance with the objective of assessing how DAO supplementation (in the form of capsules, each containing 0.3 mg DAO) can improve the symptoms associated with the condition [31]. The results of their research highlighted that DAO supplementation significantly improved all of the assessed 22 symptoms, including those affecting the cardiovascular system, the digestive tract, the skin, and the respiratory system. Similar positive results were obtained by the retrospective study of Manzotti et al., where DAO supplementation (DAO capsules were taken twice per day 15 min before lunch and dinner for at least 14 days) improved at least one of the symptoms associated with histamine intolerance in 13 out of the 14 evaluated subjects [32]. In the aforementioned study, DAO supplementation with dietary modifications led to better improvement in the quality of life of the subjects compared to diet alone. Yacoub et al. [33] and Izquierdo-Casas et al. [34] assessed how DAO supplementation can influence the symptoms that can be triggered by DAO deficiency. In their studies, 20 and 100 patients, respectively, were included. The subjects were supplemented with DAO for 30 days. The symptoms of urticaria and headache improved in the patients who received DAO. Despite DAO supplementation demonstrating promising results in these studies, most of the researchers agreed that there is a need for further research to consolidate the outcomes. This is because the sample size in these studies was limited, and there is a need to prove the results in studies of a larger scale.

Another approach to tackle histamine intolerance is using antihistamine medications [21]. However, researchers have indicated the need for further research to determine the value of this approach. This is because researchers have indicated that certain types of antihistamines, such as cimetidine and promethazine, which are H1/H2 receptor blockers, could potentially decrease DAO activity. Furthermore, the use of antihistamines is empirical, with no randomized trials to prove the value of this treatment in addressing histamine intolerance [21]. However, if necessitated, Hrubisko et al. highlighted that antihistamine use should be conscious and time-bound. Therefore, decisions related to the type of medication, dose, and treatment duration are left in the hands of the treating clinicians after considering the symptoms, their severity, and several other factors [19,21].

In addition to the approaches mentioned above, research suggests supplementing with minerals and vitamins like copper, zinc, vitamin C, and vitamin B6 in the case of known deficiency, malnutrition, or restrictive diet [19,35,36]. This is because such elements are cofactors for the DAO enzyme and should be used in conjunction with other approaches.

## 6. Challenges and Future Perspectives

Histamine intolerance comes with a unique set of challenges across various areas, like diagnosis, comorbid condition complexity, and data scarcity, among others. In this context, diagnosis remains one of the challenges that are yet to be addressed appropriately. This is because histamine intolerance is a discrete condition with non-specific symptoms that overlap with other well-established diseases [32]. One of the factors behind the difficulty in establishing a diagnosis lies in the fact that the exact mechanism behind histamine intolerance is not adequately established and documented. Moreover, certain experts consider the condition a syndrome rather than just a disease. The current diagnostic approaches lack standardization, leading to different testing techniques producing variable results, which further complicates the process of making a definitive diagnosis. In addition to the abovementioned limitation, a lack of an adopted universal definition and diagnostic algorithm endorsed by experts raises issues for research and disease management. Therefore, it is recommended that experts in the fields from various regions be gathered to develop international guidelines that can help as a starting point to create local and regional versions that better address the needs of specific populations.

Another set of challenges that are encountered with histamine intolerance is the complexity of the associated comorbid conditions [37]. In this regard, histamine intolerance is often accompanied by comorbid diseases, such as migraine and irritable bowel syndrome, among others, with heterogeneous phenotypes that fluctuate over time. With the diversity and heterogeneity of patient profiles, determining the precise contribution and interplay of histamine intolerance itself versus other involved factors is problematic. This is why approaches like dietary restriction, while they may work for some patients, cannot tackle the problem for all patients, as they do not address the root causes of the disease. This is further complicated by the inadequate understanding of histamine’s interactions within broader pathological networks, hampering efforts for developing targeted therapy for the condition alone. In addition, histamine-intolerance-specific treatment efficacy may not be adequately assessed due to the presence of comorbid conditions, which could mask the therapeutic effects.

Data scarcity is another challenge that limits progress in the domain of histamine intolerance diagnosis and management [38]. In this context, there is insufficient clinical evidence (whether publications or clinical trials). This could be attributed to various reasons, such as low disease recognition rates as well as challenges associated with establishing diagnostic criteria. This is why the currently adopted therapeutic strategies largely depend on the opinion and expertise of the treating physicians and case reports rather than high-level evidence. One robust approach to tackle this problem is utilizing prospective cohort studies that assess the condition’s natural history, identify biomarkers, and rigorously evaluate dietary and pharmacological interventions. It is important to note that while the aforementioned approach can significantly help advance the field of understanding and treating histamine intolerance, it comes with its own set of limitations, such as the need for significant resources. However, the lack of robust evidence that can help fill the current knowledge gaps justifies the time, resource, and personnel investment, which can help generate strong evidence that eventually supports optimizing our understanding of the disease and suggests better and more personalized treatment approaches rather than a one-size-fits-all approach.

In addition to the aforementioned set of challenges faced with various aspects of histamine intolerance, there exist other barriers faced by treating physicians. One such challenge is the difficulty in establishing a clear definition due to the vague and non-specific nature of the conditions [39]. This, in turn, may cause physicians to overlook the condition or clinically misdiagnose it. This not only leads to delays in addressing and treating the condition but may also add to the psychological toll that histamine intolerance has on patients. Moreover, approaches like strict dietary treatments can negatively influence the quality of life of patients and result in poor adherence. This is further complicated by the fact that there is limited specialist expertise and inadequate awareness of the condition among the public. Furthermore, the limited therapeutic choices that address histamine intolerance, as well as the use of some essential drugs that can trigger or exacerbate the condition, further complicate the treatment process.

Fortunately, the previously mentioned set of challenges can be addressed by implementing several approaches. For example, in the disease diagnosis domain, researchers and experts should work on developing a reliable set of diagnostic tests that can help differentiate histamine intolerance from closely related conditions as well as distinguish the disease in case of comorbid disorders. For example, developing neuroimaging techniques can help develop a better understanding of the connection between histamine intolerance and conditions like migraine and various others. Furthermore, assessing intestinal permeability and microbiome testing can shed a better light on how histamine intolerance overlaps with conditions such as irritable bowel syndrome. Overall, addressing comorbidities through improved diagnosis would empower patients to have better control over the symptoms of the disease and allow experts to evaluate and characterize histamine intolerance therapies more robustly.

In addition to the above, physicians should strive to establish standardized diagnostic criteria for histamine intolerance. In this context, it is essential to develop consensus diagnostic guidelines through collaboration between experts and international societies to eliminate inconsistencies and uncertainties. For example, experts need to work on developing and validating diagnostic biomarker tests that are specific for histamine intolerance. Furthermore, experts need to work on consensus criteria that would facilitate recruiting well-defined patient cohorts for studies exploring the point mentioned above.

Establishing an understanding of the pathogenic mechanisms leading to disease development is another avenue worth exploring by experts in the field. In this regard, there needs to be further research to clarify several aspects of histamine intolerance, such as mast-cell abnormalities, genetics, impairment of histamine-processing enzymes, intestinal permeability, and microbial dysbiosis. Fortunately, current omics technologies, like genomics, proteomics, and metabolomics, and the availability of tools and databases containing a breadth of information can facilitate uncovering novel disease biomarkers and drivers. This, on its own, could expedite the process of understanding the disease and establishing standards that help in its diagnosis and management. In addition, utilizing in vivo and in vitro mast-cell models may provide mechanistic insights into histamine intolerance. Collectively, elucidating pathogenesis can inform targeted therapeutic strategies and the management of comorbid conditions.

Upon the development of consensus guidelines that define diagnostic and therapeutic criteria, rigorous intervention studies need to be initiated. For example, large-scale randomized controlled trials could evaluate how dietary modifications, mast-cell stabilizers, H1/H2 receptor antagonists, antioxidants, and probiotic utilization, among other approaches, influence the disease course. The evidence generated from such studies can help further improve and fine-tune the guidelines by providing more robust evidence with tangible results.

Another aspect worth exploring is the identification of new therapeutic targets. In this context, there are several promising therapeutic targets that warrant further exploration. Examples of the latter include therapeutic agents that modulate histamine-metabolizing enzymes, intestinal barrier integrity, microbial dysbiosis, immune cell abnormalities, and histamine receptor polymorphisms. Furthermore, therapeutic agents that modulate histamine intolerance factors along with targets that affect comorbid conditions, such as treatments that regulate and influence histamine/serotonin pathways, hence affecting both the disease and related comorbidities like migraine in select patient populations, are worth exploring.

Finally, another critical area to tackle is increasing awareness among treating physicians and specialists. Local, regional, and international societies should create educational programs, support establishing specialty clinics, and develop patient guidelines to help tackle the current challenge of lack of awareness. In addition, encouraging coordinated multidisciplinary care will undoubtedly produce better patient outcomes and ultimately improve the quality of life. International collaborative networks of experts can harmonize best practices on the global stage. Overall, these efforts may result in an improved recognition of histamine intolerance, leading to a better specialist referral scheme. Such efforts can also facilitate recruitment into future clinical studies.

Collectively, addressing the aforementioned challenges with well-established and defined diagnostic approaches, elucidating pathogenic mechanisms behind histamine intolerance, conducting rigorous interventional trials to generate more robust evidence, and employing the latest trends in omics technologies to identify diagnostic and therapeutic targets, coupled with improved awareness, will undoubtedly improve the clinical management paradigm of the disease. Therefore, a collective, multidisciplinary, and wholesome approach is warranted.

## 7. Conclusions

Histamine intolerance is a complex pathophysiological condition that significantly impairs the quality of life of those affected by it. While research into this condition continues, the current evidence suggests a myriad of factors contributing to this disease, mainly involving the impaired capacity of the affected individuals to degrade histamine. Individuals affected with histamine intolerance suffer from a multitude of non-specific symptoms that make it challenging to diagnose and manage the disease effectively.

Due to limitations in objective and diagnostic tests, histamine intolerance remains a disease diagnosed through clinical exclusion. This means that further research is warranted to develop more reliable and robust biomarkers that can confirm suspected cases. In addition to the above, initiating large-scale epidemiological studies could help better characterize the true prevalence of this condition. Furthermore, a better characterization of potential environmental and genetic factors can further the understanding of the disease.

Current treatment paradigms focus on dietary modifications in the form of avoiding histamine-rich foods. Following a low-histamine diet often provides substantial relief for affected subjects. However, long-term patient adherence to this diet type poses another set of challenges. Alternative treatment approaches, such as enzyme therapy, need more rigorous clinical evaluation. Regarding histamine receptor antagonists, their efficacy needs further consolidation, which can be achieved by performing well-designed clinical trials.

Multidisciplinary care provided by an integrative medicine team, including a nutritionist, can help guide patients toward an individualized treatment regimen. A team-based approach is important given the complexity of this condition and its impact on the physical, emotional, and social well-being of affected individuals. In addition, healthcare practitioners need to be more vigilant about the condition, given its notoriety of going undiagnosed or misdiagnosed.

The better understanding and management of histamine intolerance require continued collaboration between researchers, clinicians, and patient advocacy groups. In addition, extensive, well-powered intervention studies evaluating both dietary and drug-based therapies can help establish evidence-based guidelines. Furthermore, mechanistic studies are also warranted into factors influencing histamine catabolism. A better and deeper understanding of histamine intolerance at the cellular and molecular level may reveal novel therapeutic targets.

With the growth in precision and personalized medicine, histamine intolerance can receive more attention from the research community in the upcoming years. Improved diagnostic approaches and safer and more effective therapeutic agents can significantly decrease the disease burden and provide relief to patients.

## Figures and Tables

**Table 1:** Approaches used in diagnosing histamine intolerance.

Diagnostic Approach	Comments
Serum diamine oxidase (DAO)	Intra-subject variability due to factors like time of the day limits its robustness. Thus, it should be used with other diagnostic methods to confirm the diagnosis.
Skin prick test	This test cannot differentiate between histamine intolerance and other allergic conditions. Therefore, it should solely be used to confirm the diagnosis.
Histamine challenge test	It helps determine an individual’s histamine tolerance limit. However, the value of this test is limited by challenges related to accurately quantifying histamine levels in food and beverages, the need for technical supervision from a specialist, and the emergence of reports highlighting that this test triggers symptoms in healthy individuals.
Fecal histamine levels	Accuracy is obscured due to interference from the gut microbiota, which itself produces histamine.
Genetic testing	This approach is gaining momentum and adds value when combined with other methods.

## References

[1] Tuck C.J., Biesiekierski J.R., Schmid-Grendelmeier P., Pohl D. (2019). Food Intolerances. Nutrients.

[2] Jansson-Knodell C.L., White M., Lockett C., Xu H., Shin A. (2021). High prevalence of food intolerances among US internet users. Public. Health Nutr..

[3] Crowe S.E. (2019). Food Allergy Vs Food Intolerance in Patients With Irritable Bowel Syndrome. Gastroenterol. Hepatol..

[4] Shulpekova Y.O., Nechaev V.M., Popova I.R., Deeva T.A., Kopylov A.T., Malsagova K.A., Kaysheva A.L., Ivashkin V.T. (2021). Food Intolerance: The Role of Histamine. Nutrients.

[5] Chung B.Y., Park S.Y., Byun Y.S., Son J.H., Choi Y.W., Cho Y.S., Kim H.O., Park C.W. (2017). Effect of Different Cooking Methods on Histamine Levels in Selected Foods. Ann. Dermatol..

[6] Comas-Basté O., Sánchez-Pérez S., Veciana-Nogués M.T., Latorre-Moratalla M., Vidal-Carou M.D.C. (2020). Histamine Intolerance: The Current State of the Art. Biomolecules.

[7] Izquierdo-Casas J., Comas-Basté O., Latorre-Moratalla M.L., Lorente-Gascón M., Duelo A., Vidal-Carou M.C., Soler-Singla L. (2018). Low serum diamine oxidase (DAO) activity levels in patients with migraine. J. Physiol. Biochem..

[8] Latorre-Moratalla M.L., Comas-Basté O., Bover-Cid S., Vidal-Carou M.C. (2017). Tyramine and histamine risk assessment related to consumption of dry fermented sausages by the Spanish population. Food Chem. Toxicol..

[9] García-Martín E., Ayuso P., Martínez C., Blanca M., Agúndez J.A. (2009). Histamine pharmacogenomics. Pharmacogenomics.

[10] Kucher A.N. (2019). Association of polymorphic variants of key histamine metabolism genes and histamine receptor genes with multifactorial diseases. Russ. J. Genet..

[11] García-Martín E., García-Menaya J., Sánchez B., Martínez C., Rosendo R., Agúndez J.A. (2007). Polymorphisms of histamine-metabolizing enzymes and clinical manifestations of asthma and allergic rhinitis. Clin. Exp. Allergy.

[12] Ayuso P., García-Martín E., Martínez C., Agúndez J.A. (2007). Genetic variability of human diamine oxidase: Occurrence of three nonsynonymous polymorphisms and study of their effect on serum enzyme activity. Pharmacogenet. Genom..

[13] Maintz L., Yu C.F., Rodríguez E., Baurecht H., Bieber T., Illig T., Weidinger S., Novak N. (2011). Association of single nucleotide polymorphisms in the diamine oxidase gene with diamine oxidase serum activities. Allergy.

[14] Enko D., Meinitzer A., Mangge H., Kriegshäuser G., Halwachs-Baumann G., Reininghaus E.Z., Bengesser S.A., Schnedl W.J. (2016). Concomitant Prevalence of Low Serum Diamine Oxidase Activity and Carbohydrate Malabsorption. Can. J. Gastroenterol. Hepatol..

[15] Mondovi B., Fogel W.A., Federico R., Calinescu C., Mateescu M.A., Rosa A.C., Masini E. (2013). Effects of amine oxidases in allergic and histamine-mediated conditions. Recent. Pat. Inflamm. Allergy Drug Discov..

[16] Schnedl W.J., Enko D. (2021). Considering histamine in functional gastrointestinal disorders. Crit. Rev. Food Sci. Nutr..

[17] Griauzdaitė K., Maselis K., Žvirblienė A., Vaitkus A., Jančiauskas D., Banaitytė-Baleišienė I., Kupčinskas L., Rastenytė D. (2020). Associations between migraine, celiac disease, non-celiac gluten sensitivity and activity of diamine oxidase. Med. Hypotheses.

[18] Maintz L., Novak N. (2007). Histamine and histamine intolerance. Am. J. Clin. Nutr..

[19] Kovacova-Hanuskova E., Buday T., Gavliakova S., Plevkova J. (2015). Histamine, histamine intoxication and intolerance. Allergol. Immunopathol..

[20] Schnedl W.J., Lackner S., Enko D., Schenk M., Holasek S.J., Mangge H. (2019). Evaluation of symptoms and symptom combinations in histamine intolerance. Intest. Res..

[21] Hrubisko M., Danis R., Huorka M., Wawruch M. (2021). Histamine Intolerance-The More We Know the Less We Know. A Review. Nutrients.

[22] Wöhrl S., Hemmer W., Focke M., Rappersberger K., Jarisch R. (2004). Histamine intolerance-like symptoms in healthy volunteers after oral provocation with liquid histamine. Allergy Asthma Proc..

[23] Schwelberger H.G., Feurle J., Houen G. (2013). New tools for studying old questions: Antibodies for human diamine oxidase. J. Neural Transm..

[24] Boehm T., Pils S., Gludovacz E., Szoelloesi H., Petroczi K., Majdic O., Quaroni A., Borth N., Valent P., Jilma B. (2017). Quantification of human diamine oxidase. Clin. Biochem..

[25] Pinzer T.C., Tietz E., Waldmann E., Schink M., Neurath M.F., Zopf Y. (2018). Circadian profiling reveals higher histamine plasma levels and lower diamine oxidase serum activities in 24% of patients with suspected histamine intolerance compared to food allergy and controls. Allergy.

[26] Wagner A., Buczyłko K., Zielińska-Bliźniewska H., Wagner W. (2019). Impaired resolution of wheals in the skin prick test and low diamine oxidase blood level in allergic patients. Postepy Dermatol. Alergol..

[27] Sánchez-Pérez S., Comas-Basté O., Rabell-González J., Veciana-Nogués M.T., Latorre-Moratalla M.L., Vidal-Carou M.C. (2018). Biogenic Amines in Plant-Origin Foods: Are They Frequently Underestimated in Low-Histamine Diets?. Foods.

[28] Wagner N., Dirk D., Peveling-Oberhag A., Reese I., Rady-Pizarro U., Mitzel H., Staubach P. (2017). A Popular myth—Low-histamine diet improves chronic spontaneous urticaria—Fact or fiction?. J. Eur. Acad. Dermatol. Venereol..

[29] Lackner S., Malcher V., Enko D., Mangge H., Holasek S.J., Schnedl W.J. (2019). Histamine-reduced diet and increase of serum diamine oxidase correlating to diet compliance in histamine intolerance. Eur. J. Clin. Nutr..

[30] Komericki P., Klein G., Reider N., Hawranek T., Strimitzer T., Lang R., Kranzelbinder B., Aberer W. (2011). Histamine intolerance: Lack of reproducibility of single symptoms by oral provocation with histamine: A randomised, double-blind, placebo-controlled cross-over study. Wien. Klin. Wochenschr..

[31] Schnedl W.J., Schenk M., Lackner S., Enko D., Mangge H., Forster F. (2019). Diamine oxidase supplementation improves symptoms in patients with histamine intolerance. Food Sci. Biotechnol..

[32] Manzotti G., Breda D., Di Gioacchino M., Burastero S.E. (2016). Serum diamine oxidase activity in patients with histamine intolerance. Int. J. Immunopathol. Pharmacol..

[33] Yacoub M.R., Ramirez G.A., Berti A., Mercurio G., Breda D., Saporiti N., Burastero S., Dagna L., Colombo G. (2018). Diamine Oxidase Supplementation in Chronic Spontaneous Urticaria: A Randomized, Double-Blind Placebo-Controlled Study. Int. Arch. Allergy Immunol..

[34] Izquierdo-Casas J., Comas-Basté O., Latorre-Moratalla M.L., Lorente-Gascón M., Duelo A., Soler-Singla L., Vidal-Carou M.C. (2019). Diamine oxidase (DAO) supplement reduces headache in episodic migraine patients with DAO deficiency: A randomized double-blind trial. Clin. Nutr..

[35] Rosell-Camps A., Zibetti S., Pérez-Esteban G., Vila-Vidal M., Ferrés-Ramis L., García-Teresa-García E. (2013). Histamine intolerance as a cause of chronic digestive complaints in pediatric patients. Rev. Esp. Enferm. Dig..

[36] San Mauro Martin I., Brachero S., Garicano Vilar E. (2016). Histamine intolerance and dietary management: A complete review. Allergol. Immunopathol..

[37] Smolinska S., Jutel M., Crameri R., O’Mahony L. (2014). Histamine and gut mucosal immune regulation. Allergy.

[38] Comas-Basté O., Latorre-Moratalla M.L., Sánchez-Pérez S., Veciana-Nogués M.T., Vidal-Carou M.D. (2019). Histamine and other biogenic amines in food. From scombroid poisoning to histamine intolerance. Biogenic Amines.

[39] Arih K., Đorđević N., Košnik M., Rijavec M. (2023). Evaluation of Serum Diamine Oxidase as a Diagnostic Test for Histamine Intolerance. Nutrients.

